# Group A Streptococci-Associated Necrotizing Fasciitis following Cat Bite in an Immunocompromised Patient

**DOI:** 10.1155/2017/3718360

**Published:** 2017-11-16

**Authors:** Sudheer Nambiar, Asha Karippot, Joe Devasahayam, Tony Oliver

**Affiliations:** ^1^Pulmonary Critical Care Medicine, Medical City Denton, Denton, TX, USA; ^2^Hematology and Oncology, Medical City Denton, Denton, TX, USA; ^3^Pulmonary Critical Care Medicine, University of Missouri, Columbia, MO, USA; ^4^USD Sanford School of Medicine, Vermillion, SD, USA

## Abstract

Necrotizing soft tissue infections are characterized clinically by fulminant tissue destruction, systemic signs of toxicity, and high mortality. Accurate diagnosis and appropriate treatment must include early surgical intervention and antibiotic therapy. Mortality rate is very high and could be even higher in an immunocompromised host. We present a 57-year-old female with history of rheumatoid arthritis on oral corticosteroid and methotrexate therapy with painful swelling of the left hand following a cat bite that was diagnosed as having group A streptococcus pyogenes-associated necrotizing fasciitis. Treatment with ampicillin-sulbactam, Clindamycin, and surgical debridement was performed. In spite of all the adequate therapy she succumbed to death from streptococcal toxic shock and related complications after thirty-two days of treatment in intensive care unit. Necrotizing fasciitis is an uncommon but life-threatening complication in immunocompromised hosts. Tissue infections in cat bite wounds are commonly caused by pathogenic bacterium known as* Pasteurella multocida*. Group A streptococcal infections are not reported following cat bites. A high index of suspicion must be maintained to suspect group A streptococcal associated necrotizing fasciitis following cat bites and an early medical and surgical intervention should be made for any best possible outcome.

## 1. Introduction

Necrotizing soft tissue infections include necrotizing forms of cellulitis, myositis, and fasciitis. These infections are characterized clinically by fulminant tissue destruction, systemic signs of toxicity, and high mortality [1]. Necrotizing fasciitis is an uncommon but life-threatening complication in immunocompromised hosts [2]. Group A streptococcal infections are most commonly associated with necrotizing fasciitis but are not reported following cat bites. Accurate diagnosis and appropriate treatment must include early surgical intervention and antibiotic therapy. A high index of suspicion must be maintained and an early medical and surgical intervention should be made for any best possible outcome.

## 2. Case Presentation

**Figure 1:**
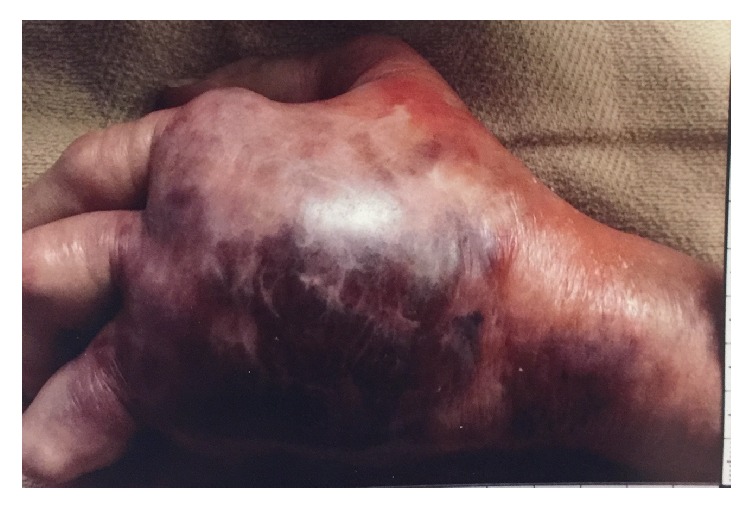
Marked swelling with discoloration of the dorsum of the left hand following cat bite.

57-year-old female with history of rheumatoid arthritis on oral corticosteroid and methotrexate therapy was admitted to the hospital for the evaluation of painful swelling of the left hand (Figure 1). She was bitten by her cat two days prior to admission. Following the bite her symptoms include fever, nausea, vomiting, and profuse diarrhea. On the day of admission she developed marked swelling with discoloration of the dorsum of the left hand. She was profoundly hypotensive and hypoxic. Examination showed elderly female in altered mental state, lethargic with bilateral upper extremity deformities from rheumatoid arthritis, with dorsum of left hand swollen with ecchymosis. Radial artery pulse was feeble. APACHE score and SOFA scores were 25 and 8, respectively. Laboratory investigations showed leukocytosis, lactic acidosis, and acute renal failure. X-ray of left hand showed diffuse soft tissue swelling of the left hand as well as sequela of chronic advanced rheumatoid arthritis of the left hand. Ultrasound of the left hand showed diffuse soft tissue swelling and lymphedema within the dorsum of the hand. Additionally there was dissecting fluid collection in the dorsal soft tissues of the hand measuring 4 cm transverse and 0.7 cm in thickness. The fluid collection appears to dissect over the second, third, and fourth metacarpals. The fluid collection lies approximately 5 mm below the skin surface.

**Figure 2:**
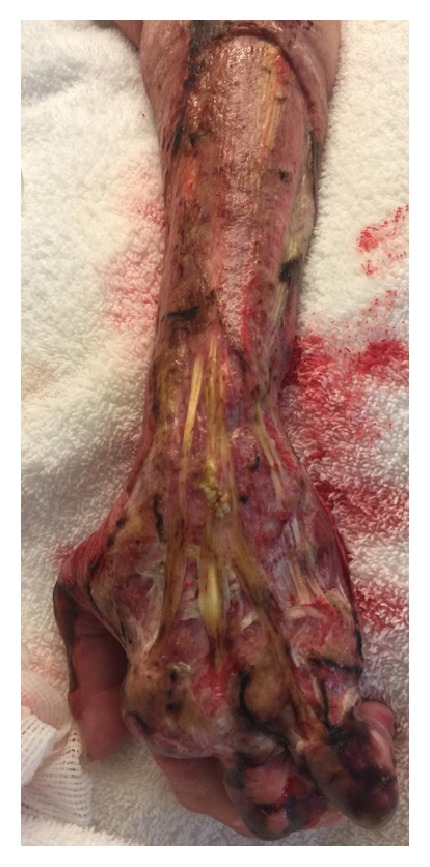
After surgical excisional debridement of skin, subcutaneous tissue, and muscle of hand and forearm.

**Figure 3:**
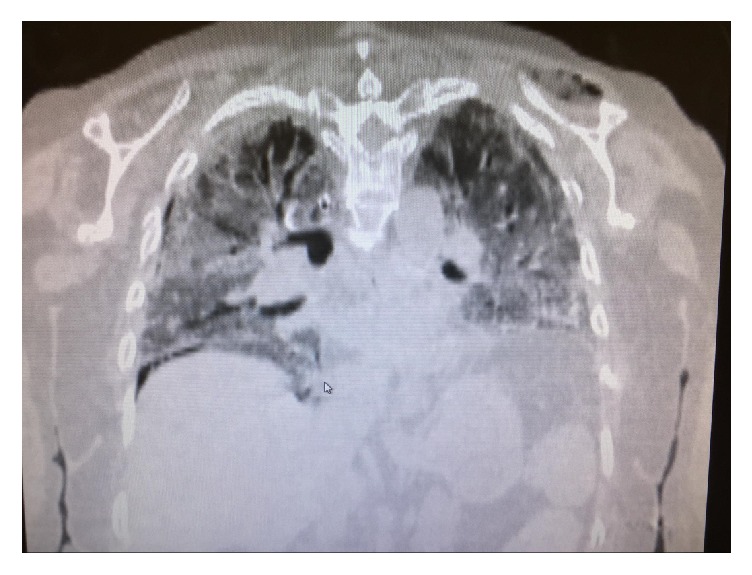
CT of chest showing bilateral infiltrates.

She was diagnosed as having necrotizing soft tissue infection of the hand and forearm. LRINEC score was calculated as 10 points. She was started on IV Penicillin G, Vancomycin, and Clindamycin. Hand surgeon was consulted and she underwent immediate surgical excisional debridement of skin, subcutaneous tissue, and muscle of hand and forearm (Figure 2). Postoperatively patient remained intubated and in hypotensive shock. A right-sided internal jugular triple lumen catheter was inserted and vasopressors and intravenous fluids were given. Infectious disease consultation was made. Intravenous Immunoglobulin was given for three days. Wound culture and blood culture grew streptococcus pyogenes sensitive to Penicillin, Clindamycin, Vancomycin, and Chloramphenicol. Vancomycin was discontinued based on culture results. With intravenous hydration the renal function improved. Patient had to undergo forearm debridement twice in the next few days by hand surgeon. Her hospital course was further complicated by acute respiratory distress syndrome [3], right femoral deep vein thrombosis, and multiple acute infarcts involving the bilateral cerebral and cerebellar hemispheres. A performed transthoracic echo confirmed the presence of Patent Foramen Ovale. She was started on subcutaneous Lovenox at 1 mg/kg body weight. She finished 21 days of IV Penicillin G for the streptococcus pyogenes and linezolid for late growth of methicillin resistant staphylococcus in the wound culture. A low tidal volume lung protective ventilator strategy was provided for the management of ARDS. In spite of all the adequate therapy for the necrotizing fasciitis, streptococcal toxic shock syndrome, and acute kidney injury, her hospital course was complicated by acute respiratory failure secondary to acute respiratory distress syndrome (Figure 3) and deep vein thrombosis with paradoxical embolism and cerebrovascular accident. Even though we followed all the recommended treatment principles such as early diagnosis, aggressive debridement, and prompt and broad antimicrobial therapy for our patient [4] our patient succumbed to death after thirty-two days in intensive care unit.

## 3. Discussion

Necrotizing soft tissue infections are characterized clinically by fulminant tissue destruction, systemic signs of toxicity, and high mortality rate. Conditions associated with necrotizing infection include diabetes, drug use, obesity, immunosuppression, recent surgery, and traumatic wounds. Group A streptococci are known to cause skin and soft tissue infections such as superficial impetigo, erysipelas, necrotizing fasciitis, myonecrosis, and toxic shock syndrome. The conditions have a gradual onset, but subsequently the process may spread rapidly with severe systemic effects and high fatality. Skin breakdown with bullae and frank cutaneous gangrene may be observed within days. The infection can progress over several hours to involve contiguous muscle groups and soft tissue resulting in necrotizing myositis. Diagnosis is usually confirmed during the surgical exploration and tissue culture results. Radiographic studies can be useful for helping determine whether muscle tissue is involved but should not delay surgical intervention when there is crepitus on examination. Vancomycin, Clindamycin, Gentamicin, and metronidazole are the initial choice of antibiotics. Antibiotic treatment should be adjusted to Gram stain, culture, and sensitivity results when available. Data evaluating the role of Intravenous Immunoglobulin in the management of necrotizing fasciitis are limited and it is not recommended. Even after early surgical intervention mortality rates of 30–40% are reported with group A streptococci-associated necrotizing fasciitis once the patient has developed septic shock.

There was one reported case in Japan about a healthy 56-year-old woman who was bitten by a cat and admitted to the hospital with fever and progressive left foot pain that was compatible with necrotizing fasciitis. Treatment with ampicillin-sulbactam and Clindamycin as well as immediate surgical debridement was performed, resulting in therapeutic success. Culture of the necrotizing tissue grew multiple organisms, including* Pasteurella multocida* and* Bacteroides caccae* [5]. Group A streptococcal infections are not reported following cat bites. Necrotizing fasciitis is an uncommon but life-threatening complication in immunocompromised hosts [2]. A high index of suspicion must be maintained to suspect group A streptococcal associated necrotizing fasciitis following cat bites and an early medical and surgical intervention should be made for any best possible outcome.

## Additional Points

*Availability of Data and Material*. From medical records.
